# Dynamic changes in GABA_A _receptors on basal forebrain cholinergic neurons following sleep deprivation and recovery

**DOI:** 10.1186/1471-2202-8-15

**Published:** 2007-02-22

**Authors:** Mandana Modirrousta, Lynda Mainville, Barbara E Jones

**Affiliations:** 1 Department of Neurology and Neurosurgery, McGill University, Montreal Neurological Institute, 3801 University Street, Montreal, Quebec H3A 2B4, Canada

## Abstract

**Background:**

The basal forebrain (BF) cholinergic neurons play an important role in cortical activation and arousal and are active in association with cortical activation of waking and inactive in association with cortical slow wave activity of sleep. In view of findings that GABA_A _receptors (Rs) and inhibitory transmission undergo dynamic changes as a function of prior activity, we investigated whether the GABA_A_Rs on cholinergic cells might undergo such changes as a function of their prior activity during waking vs. sleep.

**Results:**

In the brains of rats under sleep control (SC), sleep deprivation (SD) or sleep recovery (SR) conditions in the 3 hours prior to sacrifice, we examined immunofluorescent staining for β_2–3 _subunit GABA_A_Rs on choline acetyltransferase (ChAT) immunopositive (+) cells in the magnocellular BF. In sections also stained for c-Fos, β_2–3 _GABA_A_Rs were present on ChAT+ neurons which expressed c-Fos in the SD group alone and were variable or undetectable on other ChAT+ cells across groups. In dual-immunostained sections, the luminance of β_2–3 _GABA_A_Rs over the membrane of ChAT+ cells was found to vary significantly across conditions and to be significantly higher in SD than SC or SR groups.

**Conclusion:**

We conclude that membrane GABA_A_Rs increase on cholinergic cells as a result of activity during sustained waking and reciprocally decrease as a result of inactivity during sleep. These changes in membrane GABA_A_Rs would be associated with increased GABA-mediated inhibition of cholinergic cells following prolonged waking and diminished inhibition following sleep and could thus reflect a homeostatic process regulating cholinergic cell activity and thereby indirectly cortical activity across the sleep-waking cycle.

## Background

The basal forebrain (BF) cholinergic cells play an important role in cortical activation and arousal (see for review, [1,2]). Recently recorded and labeled using the juxtacellular technique, the cholinergic neurons discharge maximally in association with cortical activation during active waking and rapid eye movement (REM) sleep [3,4]. They cease firing in association with cortical slow wave activity during quiet, non-REM (NREM) sleep. The cholinergic cells also express c-Fos following continuous waking imposed by sleep deprivation (SD), whereas they do not express c-Fos following sleep in control (SC) or recovery (SR) conditions, comprised of >75% sleep of which ~90% is quiet, NREM sleep [5]. Their inactivity during NREM sleep could be imposed through inhibition by GABA [6,7] that can be released from co-distributed BF GABAergic neurons which discharge in association with slow wave activity [8-10] and express c-Fos in association with sleep [5].

As evidenced by increases in the amount of sleep and in the power of slow wave activity that occur following deprivation, sleep is considered to be under homeostatic control [11-13]. Such control could be determined by similar processes that serve to maintain long term stability in the excitability and activity of neurons and their circuits [14,15]. According to this homeostatic process, prolonged activity results in decreased excitability, whereas prolonged inactivity results in increased excitability. Although these changes are mediated by plastic changes in excitatory transmission [16,17], they are also importantly mediated by reciprocal, plastic changes in inhibitory transmission [15]. In cultured neurons, increased activity (stimulated by blocking K+ channels or GABA_A_Rs) results in increases in the density of GABA_A_Rs [18]. Conversely, abolition of activity (by blocking sodium channels or glutamatergic receptors) results in decreases in density of GABA_A_Rs and parallel decreases in amplitude of miniature inhibitory postsynaptic currents (mIPSCs) [18,19]. We thus envisaged that the changes in activity that occur in specific cell groups during waking and sleep could be similarly associated with dynamic changes in GABA_A_Rs and resulting inhibition.

With the knowledge that BF cholinergic neurons are active and express c-Fos during continuous waking with sleep deprivation (SD) and are inactive and do not express c-Fos during sleep with sleep control or recovery (SC or SR) (above, [5]), we investigated whether these changes in activity might be associated with changes in GABA_A_Rs. We examined immunohistochemical staining for the β_2–3 _subunits of GABA_A_Rs because immunostaining for the β_2–3 _subunits was previously shown to be present on rat cholinergic basal forebrain neurons [20] and to be altered in density or distribution on cortical neurons as a function of activity in previous *in vitro *and *in vivo *studies [19,21,22]. Moreover, mRNA for the β_3 _subunit GABA_A_R in hypothalamus was also reported to change in hypothalamus as a result of sleep deprivation in a preliminary study [23]. Across conditions of SC, SD and SR, we first examined in triple-immunostained material whether c-Fos expressing and non-expressing, choline acetyltransferase (ChAT)-immunopositive (+) cells in the magnocellular preoptic nucleus (MCPO) were immunostained for β_2–3 _GABA_A_Rs. We subsequently employed dual-immunostained material to measure the luminance of immunofluorescent staining for β_2–3 _GABA_A_Rs on ChAT+ cells across conditions.

## Results

### Sleep in SC, SD and SR conditions

As in our previous experiments [5], rats in the sleep control group (SC), which had undisturbed sleep or waking for 3 hours before sacrifice (at 1500 h), slept the majority of time (75.75 ± 0.61 %, mean ± S.E.M.); rats in the deprived group (SD) did not sleep (0 %) and remained quietly awake; and rats in the SR group that were allowed to recover sleep in the afternoon after 3 hours deprivation in the morning, slept >90% (92.87 ± 1.92 %) of the time prior to sacrifice (with a significant main effect of condition according to nonparametric, Kruskal-Wallis test statistic = 8.22, df = 2, p < 0.05). The major proportion of time for the SC and SR groups was spent in NREM sleep (68.68 ± 0.10% in SC and 80.13 ± 0.58% in SR) and a minor proportion in REM sleep (7.10 ± 0.66% in SC and 12.73 ± 1.40% in SR of total time).

### c-Fos and GABA_A_R immunostaining in cholinergic cells across conditions

**Figure 1:**
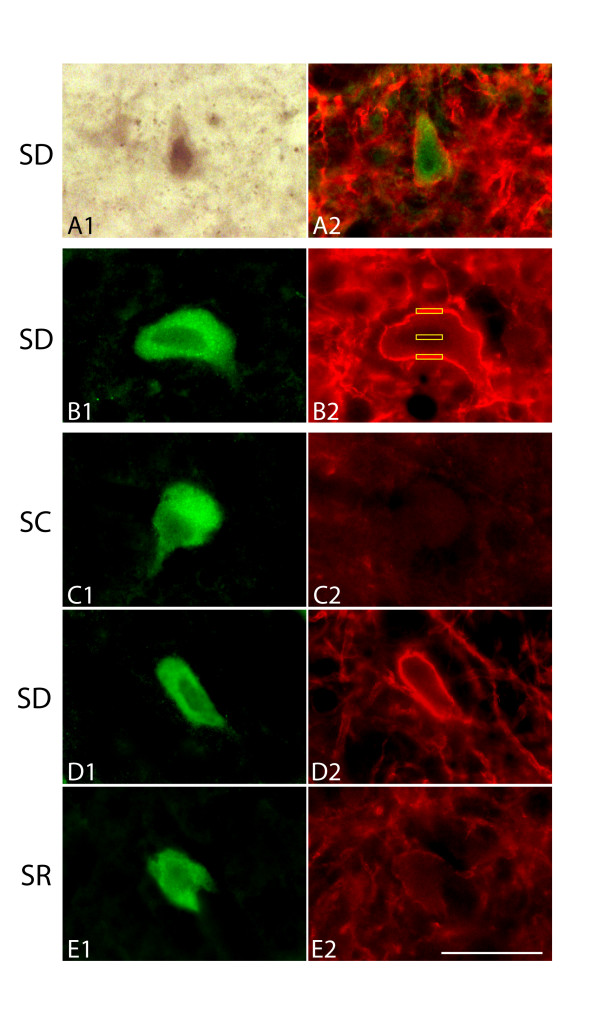
C-Fos expression and GABA_A_R labeling in BF cholinergic cells across conditions. **A**. From triple-immunostained sections, a BF cell triple-labeled for c-Fos (with DAB-Ni in gray, **A1**), ChAT (with Cy2 in green) and β_2–3 _GABA_A_R (with Cy3 in red, **A2**) from an SD brain. Note prominent GABA_A_R labeling over the plasma membrane of the cell. **B**. From dual-immunostained sections, a BF cell double-labeled for ChAT (with Cy2, **B1**) and β_2–3 _GABA_A_R (with Cy3, **B2**) from an SD brain and showing the prominent labeling along the membrane of the soma and proximal dendrites along with the boxes (in yellow) that were used for luminance measurements of the GABA_A_R labeling over the plasma membrane (on two sides) and the nucleus. The luminance of the nucleus, which was considered to represent and thus control for nonspecific, background fluorescence, was subtracted from the mean luminance of the membrane of each cell as a measure of intensity membrane GABA_A_R (Fig. 3). **C-E**. BF cells dual-immunostained for ChAT (Cy2, **C1, D1, E1**) and β_2–3 _GABA_A_R (Cy3, **C2, D2, E2**) from representative brains (selected according to mean values of membrane intensity per condition) of SC, SD and SR groups (Fig. 3). Scale bar, 20 μm. Abbreviations: SC, sleep control; SD, sleep deprived; SR, sleep recovery.

**Figure 2:**
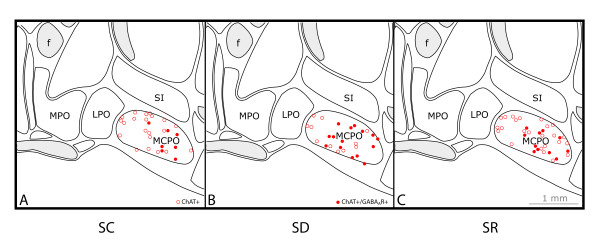
Map of BF cholinergic cells with GABA_A_R labeling across conditions. ChAT+ cells plotted in single sections (of triple-immunostained series, Fig. 1A) through the middle level of the MCPO (~A8.2) from representative brains of SC (**A**), SD (**B**) and SR (**C**) groups. Presumed to reflect a threshold for immunohistochemical detection, GABA_A_R labeling was judged to be negative (open circles) or positive (filled circles) in each ChAT+ cell within the section. Note the ChAT+/GABA_A_R+ cells are most prevalent in the SD brain. Abbreviations: f, fornix; LPO, lateral preoptic area; MCPO, magnocellular preoptic area; MPO, medial preoptic area; SI, substantia innominata.

Within sections triple-immunostained for c-Fos, ChAT and β_2–3_GABA_A_Rs, ChAT immunopositive (+) cells which expressed c-Fos were present in SD brains within the MCPO in small numbers and virtually absent in SC and SR brains, as previously reported for all BF cholinergic nuclei [5]. In the SD brains, c-Fos+/ChAT+ cells appeared positively immunostained for the β_2–3 _GABA_A_R, which was concentrated over the plasma membrane of the cell (Fig. 1A). In c-Fos-negative/ChAT+ cells in the SD and other groups, the GABA_A_R labeling was variable and particularly in the SC and SR groups not always visible, and thus presumably below the threshold for immunohistochemical detection. Using stereological random sampling and judging whether the sampled ChAT+ cells appeared either immuno-positive or -negative for β_2–3 _GABA_A_R immunostaining irrespective of c-Fos staining across groups, the double-labeled ChAT+/GABA_A_R+ cells were found to be more prevalent in the SD brains than in SC and SR brains (Fig. 2). From stereological samples and counts, the proportions of ChAT+ cells which were judged to be GABA_A_R+ differed significantly across groups (F = 7.49, df = 2, df_error _= 7, p = 0.02) and were significantly higher in the SD group (52.0 ± 0.03%) than in the SC group (20.9 ± 0.05%; according to post hoc tests with Tukey corrections for multiple comparisons, p ≤ 0.05). The proportions in the SR group (31.8 ± 0.09%) were intermediate between the SC and SD groups. This difference in receptor labeling was then assessed quantitatively in material dual-immunostained for ChAT and the β_2–3 _GABA_A_R in order to maximize the GABA_A_R staining, which was partially attenuated by the triple-immunostaining procedure used for c-Fos.

### Intensity of GABA_A_R membrane staining over cholinergic cells across conditions

**Figure 3:**
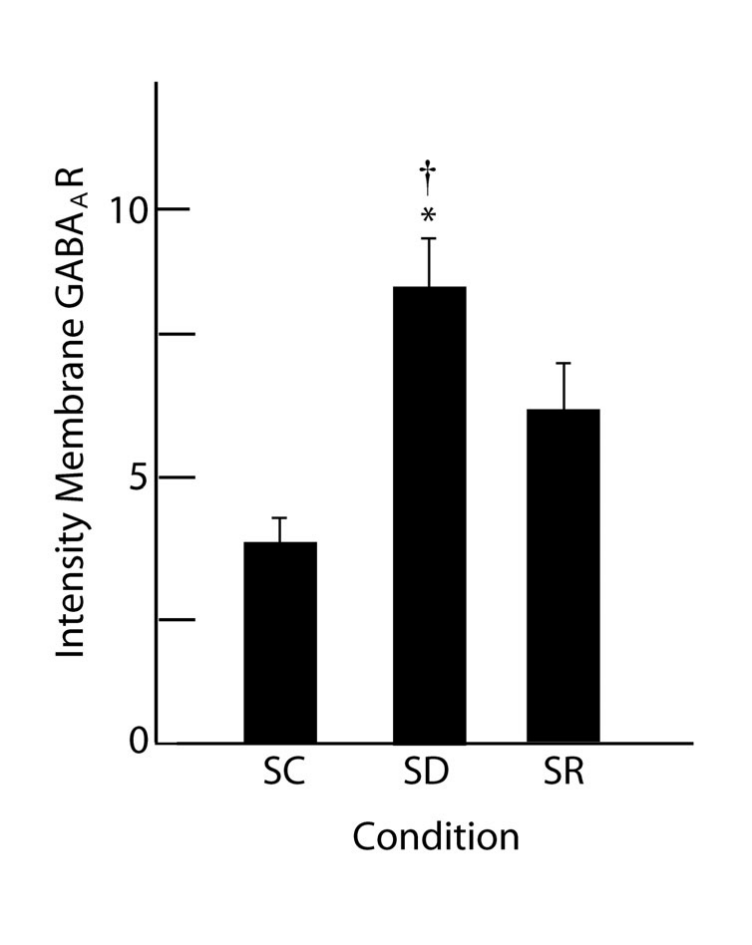
Intensity membrane GABA_A_R labeling on BF cholinergic cells across conditions. Values (mean ± SEM per group) represent luminance measures (brightness of image acquired through camera) of GABA_A_R immunofluorescence (Cy3) over the plasma membrane (average two sides) minus that over the nucleus of each ChAT+ cell sampled (see Fig. 1B). The intensity of membrane GABA_A_R labeling was significantly higher in the SD group than in the SC (*) and SR (†) groups (p ≤ 0.05, based upon Tukey adjusted post-hoc comparisons, see text).

In dual-immunostained material, the β_2–3 _GABA_A_R immunostaining was prominent over the membrane of the soma and proximal dendrites of numerous ChAT+ cells, particularly in brains from the SD group (Fig. 1B). As described in previous *in vivo *studies of GABA_A_R labeling for β_2–3_, as well as other, subunits [20,24], the fluorescent staining was commonly distributed along the full membrane of the cell body and proximal dendrites. To determine if the intensity of GABA_A_R labeling on cholinergic cells was different across SC, SD and SR conditions, luminance measures of the GABA_A_R immunofluorescence over the membrane were performed and corrected for nonspecific background fluorescence by subtracting the luminance measured over the nucleus of each cell in the acquired images (Fig. 1B2, see Methods). In randomly sampled ChAT+ cells (~25 cells per brain) obtained by stereological sampling within the MCPO (from three levels/sections per brain in 10 brains), the intensity of the membrane GABA_A_R labeling was found to differ significantly across conditions (F = 11.44, df = 2, df_error _= 224, p < 0.001; Fig. 3). It was significantly higher in the SD group than in both the SC and SR groups (p ≤ 0.05; post hoc tests with Tukey corrections for multiple comparisons).

The quantitative changes in the intensity of membrane β_2–3_GABA_A_R immunostaining were evident in the images (Fig. 1C–E) taken from ChAT+ cells having intensity measures near the average values for the SC, SD and SR groups (Fig. 3). The images illustrate how under control conditions (SC), the GABA_A_R labeling was on average very low and under the threshold for immunohistochemical detection (Fig. 1C2), how under deprived conditions (SD), it was on average very intense (Fig. 1D2), and how under recovery conditions (SR) following deprivation, it was also on average relatively low (Fig. 1E2).

## Discussion

The present results show that GABA_A_R labeling on BF cholinergic neurons increases during periods of waking when the cells are active and decreases during periods of sleep when they are inactive. They suggest that as with neurons in culture, the cholinergic neurons in the brain might undergo homeostatic regulation of their excitability as a function of prior activity through changes in GABA_A_Rs and associated inhibition across the sleep-waking cycle.

We first noted here that cholinergic cells which express c-Fos and are thus particularly active during SD [5] show membrane immunostaining for the β_2–3 _GABA_A_R. Such labeling was variable on cholinergic cells which did not express c-Fos in the SD brains and often undetectable on such cells in SC and SR brains. Across groups, GABA_A_R labeling was judged to be positive on the majority of cholinergic cells in the SD group, and only on the minority in the SC and SR groups. This labeling was considered to reflect different numbers or concentrations of GABA_A_Rs which accordingly would or would not reach threshold for immunohistochemical detection. We presume that the presence of c-Fos along with GABA_A_R labeling in neurons in the SD condition is a reflection of their prolonged activity during continuous waking. We also presume that the lack of c-Fos expression and parallel paucity of GABA_A_R labeling in the SC and SR conditions are commonly due to the amount of time spent in quiet, NREM sleep (~124 and 144/180 min on average respectively), during which the cholinergic cells would be silent [4], and not significantly affected by the amount of time spent in active, REM sleep (~13 and 23/180 min), during which the cholinergic neurons would be firing [4]. We accordingly assume that the changes in GABA_A_R labeling in the different groups reflect different levels of activity by the cholinergic neurons as predominantly high during waking in SD vs. predominantly low during sleeping in SC and SR conditions.

According to luminance measures of β_2–3 _GABA_A_R immunostaining across groups in dual-immunostained material, we found that the intensity of membrane GABA_A_R labeling was significantly greater in the SD, waking rats than in the SC, sleeping rats, suggesting that the labeling was a function of the preceding activity by the cholinergic cells. These *in vivo *results are similar to those in culture showing that prolonged increases in activity result in increased density of GABA_A_Rs along with increased inhibitory currents on hippocampal neurons [18]. Previous *in vivo *studies in the hippocampus also showed parallel increases in GABA_A_Rs and inhibitory currents following increased activity through experimentally induced seizures [21]. Here, the presumed increased activity of the cholinergic cells during 3 hours continuous waking imposed by sleep deprivation during the day would be neither as prolonged nor as extreme as that evoked in the *in vitro *and *in vivo *models, however could represent a more natural condition and resulting homeostatic adjustment to sustained activity that could occur during the natural sleep-waking cycle or disturbances to that cycle.

We also found that the intensity of membrane β_2–3 _GABA_A_R immunostaining on the cholinergic cells was significantly decreased following recovery sleep in the SR condition as compared to the SD condition. In view of the virtual silence of cholinergic neurons during NREM, slow wave sleep [4], these *in vivo *results are parallel to results in culture showing decreases in GABA_A_R labeling and inhibitory currents following abolition of activity in cortical neurons [18,19]. Although not statistically significantly different, the intensity of GABA_A_R labeling in the SR condition was not as low as that in the SC condition, possibly due to a variably incomplete return to control levels during recovery following the increase which would have occurred with the preceding 3 hour deprivation.

In the immunofluorescent images, we found that the β_2–3 _GABA_A_R immunostaining was concentrated over the plasma membrane of the cell, often distributed along the entire membrane of the soma and proximal dendrites and altered in intensity over the membrane as a function of condition. This relatively continuous, though neither homogeneous nor clustered, pattern of *in vivo *staining for the β_2–3_, as well as other, subunit GABA_A_Rs has been described previously for *in vivo *staining of basal forebrain as well as other neurons in the brain [20,24]. Such membrane staining is presumed to reflect functional receptors, which would contain β subunits [21]. The continuous distribution likely reflects the presence of many, including the β_2–3 _subunit, GABA_A_Rs in the extrasynaptic as well as synaptic membrane of the cells [25-27]. Both extrasynaptic and synaptic membrane receptors nonetheless reflect functional GABA_A_Rs, which respectively mediate tonic and phasic currents [28]. Accordingly, the changes in intensity of the β_2–3 _GABA_A_R immunostaining over the membrane observed in the present study could reflect changes in functional receptors. Increases in membrane GABA_A_Rs would be associated with marked enhancement of inhibition since the GABA_A_Rs in the central nervous system are believed to be commonly fully saturated by the release of GABA from one synaptic vesicle [29]. Indeed, the number of surface β_2–3 _subunit GABA_A_Rs has been shown to be directly correlated with the amplitude of inhibitory currents [21,22]. Although it cannot be surmised how the changes in membrane GABA_A_Rs occurred in the present study, it is known that rapid increases in cell surface GABA_A_Rs can occur by their recruitment to the membrane from intracellular stores [21,22]. Reciprocally, rapid decreases can occur by internalization or endocytosis of the receptors [30,31]. Given preliminary reports of increases in mRNA for β_3_GABA_A_Rs following 7 hours of sleep deprivation and varying levels of the mRNA in relation to the sleep cycle [23], it is also possible that the changes in membrane GABA_A_Rs involve changes in protein synthesis. Indeed, increases or decreases in membrane GABA_A_Rs can occur through multiple mechanisms involving differential intracellular trafficking along with changes in protein synthesis, phosphorylation and degradation [30,31].

## Conclusion

According to our results and interpretations, membrane GABA_A_Rs on the BF cholinergic neurons would progressively increase as a function of activity during waking and progressively decrease as a function of inactivity during NREM, slow wave sleep. Since the cholinergic neurons stimulate fast gamma activity and attenuate slow delta activity on the cortex [1,32,33], an increase in GABA-mediated inhibition of the cholinergic cells following waking would be associated with decreases in fast and increases in slow cortical activity, and a decrease in GABA-mediated inhibition during sleep would be associated with reciprocal increases in fast and decreases in slow cortical activity. Such hypothetical changes parallel those measured in slow, delta activity which varies as a function of prior waking, being maximal at the onset of sleep and decreasing progressively during sleep [12]. The dynamic changes in GABA_A_Rs on cholinergic BF neurons would thus reflect as well as participate in the homeostatic regulation of cerebral activity across the sleep-waking cycle.

## Methods

All procedures were approved by the McGill University Animal Care Committee and conformed to the Canadian Council on Animal Care.

### Sleep deprivation

Male Wistar rats (average ~240 g at termination of experiment, corresponding to ~50 days old) were housed individually with free access to food and water at all times with a 12:12 light/dark schedule (lights on from 700 to 1900 h). As previously described [5], the rats were deprived of sleep and observed in their home cages so as to avoid stress. All rats were sacrificed at 1500 h and were previously submitted respectively to 1) total sleep deprivation (SD) for 3 hours (1200 to 1500 h, n = 3), 2) total sleep deprivation for 3 hours (900 to 1200 h) followed by sleep recovery (SR) for 3 hours (1200 to 1500 h, n = 3), or 3) undisturbed sleep and waking as sleep control (SC) for 3 hours (1200 to 1500 h, n = 4). Rats in the SD and SR groups were deprived of sleep by being gently touched with a paint brush upon closure of their eyes. Sleep-wake states were scored every 20 sec by behavioral observations as wake, NREM sleep or REM sleep. Behavioral scoring was used in order to avoid the stress of surgery and tethering for recording and has been found to be sufficient for scoring the major states of wake, NREM (behaviorally quiet) sleep and REM (behaviorally active with twitches) sleep in normal rats [5,34,35]. At the end of the experiment (1500 h), rats were immediately killed under pentobarbital anesthesia (100 mg/kg, i.p.) by intra-aortic perfusion with a fixative solution of 3% paraformaldehyde.

### Immunohistochemistry

Following immersion in a 30% sucrose solution, brains were frozen and stored at -80°C. They were cut in coronal sections at 20 μm thickness and collected at 800 μm intervals as multiple series through the basal forebrain. One series of sections from each brain (n = 10) was immediately processed for triple immunohistochemical staining of c-Fos, ChAT and GABA_A_R. The remaining series were frozen in 30% glycerol-ethylene glycol solution and stored at -20°C. Following stereological analysis (below) of the triple-immunostained series which revealed changes in GABA_A_R labeling of ChAT+ cells, one series of sections from all brains (n = 10) were simultaneously processed for dual immunofluorescent staining for ChAT and GABA_A_R in order to maximize the receptor labeling and allow for quantitative measures of the labeling using luminance measures (below).

Antibodies were employed for triple or dual immunohistochemical staining for c-Fos, ChAT and GABA_A_R. For c-Fos, a rabbit (Rb) antiserum (1:10,000, Ab-5, PC38, Oncogene Research Products, San Diego, CA) was employed and revealed using the peroxidase-anti-peroxidase (PAP) technique (Rb PAP and donkey (Dky) anti-Rb from Jackson ImmunoResearch Laboratories, West Grove, PA) processed with diaminobenzidine-nickel (DAB-Ni). For ChAT, a Rb antiserum (1:1000, AB143, Chemicon International, Temecula, CA) was employed and revealed by immunofluorescent staining with Dky Cy2-conjugated anti-Rb antiserum (Jackson). For the GABA_A_R, a mouse (Ms) monoclonal antibody against the β_2–3 _chain subunits (1:100, MAB341, Chemicon) was employed and revealed using immunofluorescent staining with Dky Cy3-conjugated anti-Ms antiserum (Jackson). For the triple-immunostained series, the sections were first incubated with the c-Fos antibody overnight at room temperature and processed by PAP staining with DAB-Ni, which produced black staining over the nucleus. For both triple- and dual-immunostained series, the sections were co-incubated with ChAT and GABA_A_R antibodies for three nights at 4°C followed by co-incubation with Cy2- and Cy3-conjugated secondary antisera for 2 hours. The ChAT Cy2 staining was seen within the cytoplasm, and the GABA_A_R Cy3 staining over the plasma membrane (see Results).

### Microscopic analysis

Sections were viewed by light and fluorescence microscopy with a Nikon Eclipse E800 microscope equipped with an x/y/z movement-sensitive stage and digital camera (Optronics, Microfire S99808, Goleta, CA) attached to a computer. Fluorescence was viewed and acquired using appropriate filter sets for Cy2 (bandpass excitation filter, 460–500 nm; longpass dichromatic mirror, 505 nm; bandpass emission filter, 510–560 nm) and Cy3 (bandpass excitation filter, 510–560 nm; longpass dichromatic mirror, 565 nm; longpass emission filter, 590 nm). Cell counts and image acquisition were performed by systematic random sampling using StereoInvestigator (MicroBrightField, MBF, Williston, VT). On the acquired images, measurements were performed for brightness of the fluorescence, thus called luminance since it refers to the brightness of the light transmitted through the camera, using Neurolucida software (MBF). For these applications, a computer resident atlas was employed through the rat BF (~Anterior, A7.0 to A11.0 from interaural zero) [5].

### Cell counts

In triple-immunostained series, ChAT+ cells were examined for being positively labeled for the β_2–3 _GABA_A_R and for c-Fos (Fig. 1A). Single-, double- and triple-labeled cells were plotted and counted in all brains (n = 10) through the MCPO at three levels (corresponding to three sections from the rostral to caudal extent of the nucleus at ~A9.0, A8.2 and A7.4) using the Optical Fractionator program of StereoInvestigator to provide unbiased sampling and resulting estimates of proportions of labeled cells through the nucleus. Within the stereology program, cells were counted under a 60× oil objective (with 1.4 numerical aperture) using a counting frame of 125 × 125 μm, which yielded 3 or more cells counted per frame, and sampling grid of 250 × 250 μm, which yielded 10 counting sites or more per section and >35 counting sites for the MCPO per brain. In one representative brain from each series, cells were mapped and counted using a sampling grid of 125 × 125 μm for representation of all cells per section. Cells that came into focus beneath the surface of each section were counted within a counting block of 8 μm in depth (in the mounted and dehydrated sections that were on average 10 μm thick).

### Luminance measurements

In dual-immunostained series, images of ChAT+ cells and their β_2–3 _GABA_A_R immunofluorescence were acquired using the 8-bit setting of the digital camera, which thus provides a gray scale of 0–256 levels for luminance measures in Neurolucida. For all images, the fluorescence illumination was the same, and the settings of the camera were the same (gain of 4 and 100 ms exposure). Image acquisition was made as rapidly as possible for each cell so as to avoid bleaching of the fluorescence. In each brain (n = 10), random sampling through three levels (corresponding to three sections at ~A9.0, A8.2 and A7.4) of the MCPO was performed using StereoInvestigator. Within the Optical Fractionator program, a counting frame of 50 × 50 μm and grid of 250 × 250 μm were employed for sampling ~25 sites and thus cells per brain. In each site, the ChAT+ cell which was the closest to the centre of the counting frame was selected for image acquisition. The images of the ChAT+ cell and its GABA_A_R labeling were acquired using a 60× oil objective (Fig. 1B–E). Luminance measurements were performed on the acquired fluorescent images using Neurolucida, which provides data in arbitrary units (on a gray scale of 0 – 256). For this application, a box was created with a length of 5 μm and a width of 1 μm, which was established as the maximal length and thickness of GABA_A_R staining that could be consistently measured over the plasma membrane of the ChAT+ cells. In each image, boxes were positioned parallel to the long axis of the cell soma over the plasma membrane (on two sides) and over the nucleus (Fig. 1B2) for collection of average luminance values within each box. The average luminance values (mean ± SEM for measurements over 227 cells in 10 brains) were 49.18 ± 0.88 for the plasma membrane and 45.10 ± 0.81 for the nucleus. For each cell, the intensity of membrane GABA_A_R immunostaining was calculated by taking the average membrane luminance (of the two sides) and subtracting the luminance of the nucleus, which was considered to represent nonspecific immunofluorescence and thus by subtraction to control for variations in background staining across cells, sections and brains.

### Statistical analysis and presentation

Data were analyzed across conditions by the nonparametric Kruskal-Wallis test for sleep-wake values, which included zero values, or by one way ANOVA followed by Tukey corrected post-hoc comparisons for cell counts and luminance measures (Systat, v10.2, Richmond, CA). Figures were composed using Adobe Photoshop and Illustrator Creative Suite (Adobe, San Jose, CA). Images are presented as they were acquired using the 8-bit setting of the camera which is compatible with the 8-bit image option of Adobe Photoshop and without applying any adjustment for brightness or contrast.

## Abbreviations

BF, basal forebrain

ChAT, choline acetyltransferase

GABA_A_R, GABA_A _receptor

MCPO, magnocellular preoptic area

mIPSC, miniature inhibitory postsynaptic current

NREM, nonREM

REM, rapid eye movement

SC, sleep control

SD, sleep deprived

SR, sleep recovery

## Authors' contributions

MM conducted all the experiments, analyzed the data and drafted the manuscript; LM provided technical assistance; BEJ directed the experiments, reviewed all data analysis and wrote the final manuscript. All authors read and approved the manuscript.
